# Effect of minocycline on changes in affective behaviors, cognitive function, and inflammation in breast cancer survivors undergoing chemotherapy: a pilot randomized controlled trial

**DOI:** 10.1007/s10549-024-07457-w

**Published:** 2024-08-14

**Authors:** Zihan Melink, Maryam B. Lustberg, Patrick M. Schnell, Jessica Mezzanotte-Sharpe, Tonya S. Orchard

**Affiliations:** 1 Department of Human Sciences, The Ohio State University, Columbus, OH 43210 USA; 2 Yale School of Medicine, Center for Breast Cancer, New Haven, CT 06511 USA; 3 Division of Biostatistics, College of Public Health, The Ohio State University, Columbus, OH 43210 USA; 4 Division of Hematology/Oncology, Department of Medicine, Vanderbilt University Medical Center, Nashville, TN 37232 USA

**Keywords:** Minocycline, Depression, Anxiety, Affective disorders, Breast cancer, Chemotherapy

## Abstract

**Purpose:**

Minocycline suppresses chemotherapy-induced neuroinflammation in preclinical models, but its effects in cancer survivors are unknown. This study evaluated the longitudinal effects of minocycline on affective behaviors, cognitive functions, and inflammation in women with breast cancer (BC) undergoing chemotherapy.

**Methods:**

This is a pilot, double-blind, randomized controlled trial of oral minocycline (100 mg BID) versus placebo for chemotherapy-induced affective disorders in women initiating chemotherapy for stage I–III BC. Participants received minocycline or placebo up to one week before chemotherapy, continuing through cycle 4 (C4). Epidemiologic Studies Depression Scale (CES-D) and State-Trait Anxiety Inventory (STAI) were assessed at baseline, each cycle of chemotherapy (C1–C4), 2–3-week post-chemotherapy (end of chemotherapy), and 6-month post-chemotherapy (6 M) as the primary outcomes. Sub-group analysis of CES-D and STAI based on the severity of symptoms was also performed. Changes in self-reported cognition and serum inflammatory markers were also evaluated.

**Results:**

Fifty-seven women enrolled and 55 completed the study. Except for Interleukin-8 (*p* ≤ 0.03), changes in inflammatory markers, cognitive function, CES-D, and STAI were not significantly different between groups from baseline to any cycle or post-chemotherapy time point (all *p* > 0.05), adjusting for baseline scores. Increases in serum Interleukin-8 from baseline to C4 and 6 M were ameliorated by minocycline (*p* < 0.05). The sub-group symptomatic for depression (CES-D > = 16 at baseline) treated with minocycline had a greater reduction in CES-D score compared to placebo from baseline to 6 M (*p* = 0.01).

**Conclusion:**

Despite attenuation of IL-8, minocycline did not alter self-reported affective symptoms or cognition in this cohort of BC survivors undergoing chemotherapy. The effect of minocycline on BC survivors symptomatic for depression before chemotherapy warrants further investigation.

**Supplementary Information:**

The online version contains supplementary material available at 10.1007/s10549-024-07457-w.

## Purpose

Approximately 4.1 million women live with breast cancer (BC) and nearly 38% of people with cancer are diagnosed with clinically significant levels of anxiety and depression within the first five years after the cancer diagnosis [1, 2]. Importantly, these symptoms can persist for years [1, 3], impacting adherence to cancer treatment and, therefore, the survival rate [1, 4]. Depression and anxiety adversely affect cancer recurrence, survival, and mortality in both breast cancer and other cancer populations based on current meta-analysis [5, 6]. Additionally, symptoms of anxiety and depression have been associated with other negative consequences of chemotherapy, such as cancer-related cognitive impairment (CRCI) [7–10].

Almost two-thirds of BC patients receive multi-agent chemotherapy, such as doxorubicin (DOX) plus cyclophosphamide or the more recently used taxane-based chemotherapy regimens [11, 12]. Anthracycline regimens such as DOX plus cyclophosphamide have been associated with anxiety-like and depressive-like behavior, as well as cognitive impairment, in animal models of chemotherapy [13–16]. Taxane-based chemotherapies have been shown to worsen emotional distress, including symptoms of depression, anxiety, and cognitive impairment in women treated for BC [12, 17–19]. Nearly three-fourths of patients with non-central nervous system tumors complain of CRCI post-chemotherapy [19–21].

Preclinical and clinical studies have revealed several potential underlying biologic mechanisms of cancer and cancer therapy-induced affective symptoms, including microglial activation, elevated inflammation, neurotransmitter metabolism dysregulation, and disrupted neurogenesis [13–16]. Microglia are immune cells of the brain that, when activated, release pro-inflammatory cytokines and reactive oxygen species [20]. Doxorubicin induces microglial activation and has been associated with increased depressive-like behavior in animal models [21, 22]. The DOX regimen has been shown to increase oxidative stress and neuroinflammation, potentially providing a mechanism through which CRCI can occur [14–16]. Although not fully understood, some proposed underlying mechanisms for Taxane-induced affective and cognitive dysfunction include loss of spines and dendritic arborization, loss of cortical gray matter, impaired neurotransmission, and destabilized neuronal structures [17, 23].

Minocycline is a second-generation, broad-spectrum tetracycline antibiotic that crosses the blood–brain barrier and has been shown to inhibit microglial activation and exert anti-inflammatory and neuroprotective properties in many preclinical and clinical studies [24–34]. Furthermore, studies have shown that minocycline can help reduce anxiety-like behaviors and alleviate depressive symptoms in people with major depressive disorder ([35, 36] as cited in [37]). However, little research has been done to explore the effect of minocycline on symptoms of depression and anxiety in women with BC.

We hypothesized that minocycline could reduce adverse symptoms of depression, anxiety, and cognitive impairment. Due to minocycline’s effectiveness in other populations, our primary hypothesis refers to the overall effect rather than one specific to a particular mechanism of action. However, based on promising preclinical data [25, 26, 30, 34], we hypothesized that chemotherapy activates microglia, resulting in inflammation in women being treated for BC, thus precipitating or exacerbating affective disorders like anxiety and depression and contributing to cognitive changes as well as other adverse outcomes. Further, we hypothesized that minocycline, an immunomodulator, can attenuate microglial activation, thereby reducing inflammation. Therefore, this study examined the oral administration of minocycline during neoadjuvant or adjuvant chemotherapy and the effect on symptoms of depression, anxiety, cognitive changes, and inflammatory markers in breast cancer patients. The study was not powered for a full analysis of inflammation as a mediator of the effect of minocycline on the primary outcomes.

## Methods

### Eligibility

Women over the age of 18 were eligible for the study if they were diagnosed with stage I–III breast cancer and were initiating first-line adjuvant or neoadjuvant chemotherapy. Patients were excluded from the study if they had any of the following: rheumatoid arthritis or any other autoimmune or inflammatory joint disease (with the exception of osteoarthritis and fibromyalgia), known bleeding disorders, second malignancy or any metastatic malignancy, current use of anticoagulation therapy, pregnancy, breastfeeding, tetracycline allergy, the inability to give informed consent, and any uncontrolled intercurrent illness that would limit compliance with study requirements, including but not limited to active or ongoing infection, symptomatic congestive heart failure, unstable angina pectoris, cardiac arrhythmia, or psychiatric illness or social situation that would limit compliance with the study requirements.

### Study design

This was a pilot double-blinded randomized study of minocycline (100-mg BID) vs. matched placebo tablets (BID) for cancer-related affective behaviors in women receiving chemotherapy for breast cancer. Eligible participants were women with BC who underwent treatment at the Stefanie Spielman Comprehensive Breast Center. Informed consent was obtained from those who met the study criteria (see inclusion/exclusion criteria). Participation in the study did not affect the dose or the timing of subsequent chemotherapy treatments. Consented participants were randomized to either oral administration of minocycline or placebo with matched appearance for up to a 1-week loading period before chemotherapy, then concurrent administration during the chemotherapy treatment period (i.e., through cycle 4), and an optional subsequent two-week period following chemotherapy. The optional period was offered due to the potential for chemotherapy agents to remain in the body for several weeks post-treatment [38, 39].

### Affective behavior measurements

Primary outcome measures of depression and anxiety were assessed using CES-D and STAI. The assessments were performed at baseline, cycle (C) 2, C3, C4, end of chemotherapy, and at six-month post-treatment (6 M). The STAI, established by CD Spielberger in 1983, evaluates feelings of tension, nervousness, worry, and apprehension and has been used frequently in studies to examine negative affective symptoms [40]. STAI was widely used to measure anxiety in cancer survivors [41–43]. A lower STAI score indicates less symptomatology and a score higher than 39 suggests symptoms of anxiety [41, 44]. The CES-D, established by LS Radloff in 1977, is a twenty-item checklist and lower scores suggest lesser depressive symptoms. It has been validated in different ethnic groups and is commonly used in assessing depressive symptoms [45, 46]. Similarly, CES-D has been used widely to assess depressive symptoms in cancer patients [47]. The common cut point for CES-D is 16, where a score higher than 16 suggests the test subject is symptomatic [47–49].

### Cognitive measurements

Two self-reported assessments, Behavior Rating Inventory of Executive Function-Adult Version (BRIEF A) and Multifactorial Memory Questionnaire (MMQ), were collected at baseline, end of chemotherapy, and six-month post-treatment (6 M) to probe the cognitive changes in the subjective aspects of cognitive function. The BRIEF A, published by RM Roth in 2005, is a self-report of executive functioning previously used in BC patients to examine complaints in memory, organization, task monitoring, and overall global executive function [50]. BRIEF A is composed of two indices, the Behavioral Regulation Index (BRI) and the Metacognitive Index (MI) [51]. The BRI index includes four subscales, including Inhibit, Shift, Emotional control, and Self-monitoring, which capture the ability to maintain appropriate regulatory control of one’s own behavior and emotional responses [51]. The MI index contains five subscales, including Initiate, Working Memory, Plan/Organize, Task Monitor, and Organization of Materials, and reflects the individual’s problem-solving abilities [51]. The sum of the two sub-indices yields an overall score (Global Executive Composite, GEC) [51]. For the total score GEC and the sub-indices MI and BRI, lower scores suggest better cognitive and executive functions [51]. MMQ is a self-report questionnaire for assessing multiple subjective aspects of the working memory [52]. MMQ also has three sub-indices: MMQ-satisfaction (also known as feelings sub-index), which probes the participant’s worry and contentment about memory; MMQ-ability (also known as mistakes sub-index), which measures subjective forgetfulness; and MMQ-strategy, which measures compensatory strategies [52]. Higher MMQ-satisfaction and MMQ-ability scores and lower MMQ-strategy scores suggest better memory performance [52, 53].

### Inflammatory biomarkers

Peripheral inflammatory markers, including serum cytokines and one cytokine receptor, were measured using the Meso QuickPlex SQ 120 (Meso Scale Discovery, 1601 Research Boulevard, Rockville, MD.) at the Ohio State University Clinical Research Center by the electrochemiluminescence method. No blood was drawn for this study at the end of chemotherapy visit, therefore the three timepoints of measurement were baseline, C4, and 6 M post-chemotherapy. The lower limit of detection (LLOD) for inflammatory markers were 0.05 pg/ml for Interleukin 1 beta (IL-1b), 0.06 pg/ml for Interleukin-6 (IL-6), 0.07 pg/ml for Interleukin-8 (IL-8), 0.04 pg/ml for Tumor Necrosis Factor-alpha (TNFa), and 0.2 pg/mL for TNF-α receptor 2 (TNF-RII). The intra-assay coefficients of variation were 3.8%, 4.0%, 3.1%, 2.8%, and less than 10–20%, respectively, for the above markers.

### Statistical analysis

Drug compliance was evaluated using pill counts and MEMS caps. Intention-to-treat (ITT) populations were used for safety and compliance measures analysis. We used a quasi-Poisson generalized linear model to test for a difference in missed dose counts between randomized arms at each cycle.

Two Sample *t* tests were planned to measure the differences in CES-D and STAI scores by randomization arms at each time point. Because significant differences were observed at baseline, the analysis was adjusted for baseline scores using linear mixed models fit by Restricted Maximum Likelihood (REML).

The changes in affective behavior and cognitive assessments over time from baseline to the end of the study were evaluated for each of the groups, as were the differences in the changes between the two groups after four cycles of chemotherapy (for inflammatory markers) and at end of chemotherapy (for primary and other secondary outcomes). Mixed models for repeated measures were used to account for the association of measures over time from the same patients.

### Adverse events

The Common Terminology Criteria for Adverse Events v4.0 (CTCAE) of the National Cancer Institute was used to define adverse events. The severity of adverse reactions was categorized as grade 1 to grade 5 in increasing severity. Grade 3, 4, and 5 toxicities were reported as serious adverse events. Adverse events were attributed as unrelated, unlikely to be related, possibly related, probably related, or definitely related to treatment by evaluators blinded to treatment assignment.

## Results

### Participant characteristics

A total of 57 participants enrolled in the study and were randomized into minocycline and placebo groups. One participant withdrew after the first cycle of chemotherapy and the other after the fourth cycle of chemotherapy. Fifty-five women completed the study and 54 participants completed the primary measurements. Participants took the study drugs during the time of their chemotherapy treatment (i.e., C1–C4) which ranged from 42 to 72 days, with a mean (SD) of 54.6 (10.8) days of treatment. None of the participants chose to continue the study drugs during the optional period two weeks after Cycle 4. The majority (98.2%) of patients in this study identified as non-Hispanic white. 47.3% participants were ER+/PR+, HER2−, 27.3% were HER+, and 12% were triple negative. The stage distribution was as follows: 29.1% I, 56.4% II, and 14.5% III. The median age was 52 years (range 26–71). Twenty-six patients (47.3%) received an anthracycline (AC)-containing regimen, while 28 (50.9%) received a non-AC-containing regimen. Additional demographics are shown in Table 1.

**Table 1:** Baseline demographics of 55 women randomized to minocycline or placebo

	Minocycline (*N* = 28)	Placebo (*N* = 27)	Overall (*N* = 55)
*Age*			
Mean (SD)	50.9 (11.0)	51.8 (11.0)	51.3 (10.9)
Median [Min, Max]	52.0 [29.0, 71.0]	52.0 [26.0, 71.0]	52.0 [26.0, 71.0]
*Gender*			
F	28 (100%)	27 (100%)	55 (100%)
*Race*			
Asian	1 (3.6%)	0 (0%)	1 (1.8%)
Black or African American	1 (3.6%)	3 (11.1%)	4 (7.3%)
White	26 (92.9%)	24 (88.9%)	50 (90.9%)
*Ethnicity*			
Hispanic or Latino	1 (3.6%)	0 (0%)	1 (1.8%)
Non-Hispanic	27 (96.4%)	27 (100%)	54 (98.2%)
*Receptor summary*			
ER+/PR+, HER2−	12 (42.9%)	14 (51.9%)	26 (47.3%)
HER2+	9 (32.1%)	6 (22.2%)	15 (27.3%)
Triple−	6 (21.4%)	6 (22.2%)	12 (21.8%)
Unknown	1 (3.6%)	1 (3.7%)	2 (3.6%)
*Performance status*			
0—Fully active	26 (92.9%)	25 (92.6%)	51 (92.7%)
1—Restricted	2 (7.1%)	0 (0%)	2 (3.6%)
Missing	0 (0%)	2 (7.4%)	2 (3.6%)
*Stage*			
I	9 (32.1%)	7 (25.9%)	16 (29.1%)
II	15 (53.6%)	16 (59.3%)	31 (56.4%)
III	4 (14.3%)	4 (14.8%)	8 (14.5%)
*Nodes*			
0	5 (17.9%)	4 (14.8%)	9 (16.4%)
1	5 (17.9%)	1 (3.7%)	6 (10.9%)
2	2 (7.1%)	0 (0%)	2 (3.6%)
4	0 (0%)	2 (7.4%)	2 (3.6%)
Missing	16 (57.1%)	20 (74.1%)	36 (65.5%)
*Chemotherapy type*			
No chemotherapy	1 (3.6%)	0 (0%)	1 (1.8%)
Anthracycline	12 (42.9%)	14 (51.9%)	26 (47.3%)
Non-anthracycline	15 (53.6%)	13 (48.1%)	28 (50.9%)

### Compliance

Participants were expected to take study drugs twice a day up to one week prior to start of chemotherapy and through the duration of chemotherapy. Duration of chemotherapy varied based on the specific regimen and patient experiences, with a range of 45–93 days and a mean (SD) of 68(14) days of treatment. There were no statistically significant differences in doses missed between randomized groups during any cycle (Supplementary Fig. 1, *p* = 0.21–0.59). The median dosage missed during Cycle 1 is 2 for the minocycline group and 1 for the placebo group. The maximum dosage missed during Cycle 1 was 41 for the minocycline group and 27 for the placebo group. The median dosage missed during Cycle 2 for both arms was 1, and the maximum dosage missed was 19 for the minocycline group and 41 for the placebo group. The median dosage missed during Cycle 3 is 0.5 for the minocycline group and 2 for the placebo group. The maximum dosage missed during Cycle 3 is 16 for the minocycline group and 24 for the placebo group. The median dosage missed during Cycle 4 is 1 for the minocycline group and 1.5 for the placebo group. The maximum dosage missed during Cycle 4 is 35 for the minocycline group and 38 for the placebo group. Overall, 16 participants had complete pill count data for all four time points (Cycle 1–Cycle 4). Of the 16 participants, 15 (93.75%) took > 80% of expected doses.

### Adverse events

Adverse events by randomization arms are shown in supplementary data (Supplementary Table 1). The only statistically significant difference in adverse events between randomized arms was a lower incidence of diarrhea in the minocycline arm (28.6% versus 64.3%, *p* = 0.02), which remained so when analysis was restricted to events attributed as possibly, probably, or definitely related to treatment (7.1% versus 32.1%, *p* = 0.04). Of 698 adverse events (allowing multiple events of the same type per participant) reported, 83% were judged unrelated or unlikely to be related. There was no statistically significant association between attribution and randomized group (Chi-squared test *p* = 0.1). There were three Grade 3 adverse events possibly, probably, or definitely related to treatment: diarrhea (“probably” on placebo, “possible” on minocycline) and an eye disorder (“possible” on placebo). There were three Grade 4 adverse events of any attribution: decreased lymphocyte count (“unrelated”), sepsis (“unrelated”), and hyperglycemia (“unlikely”), all in the placebo group.

### Mean scores and changes in affective behavior scores

**Fig. 1:**
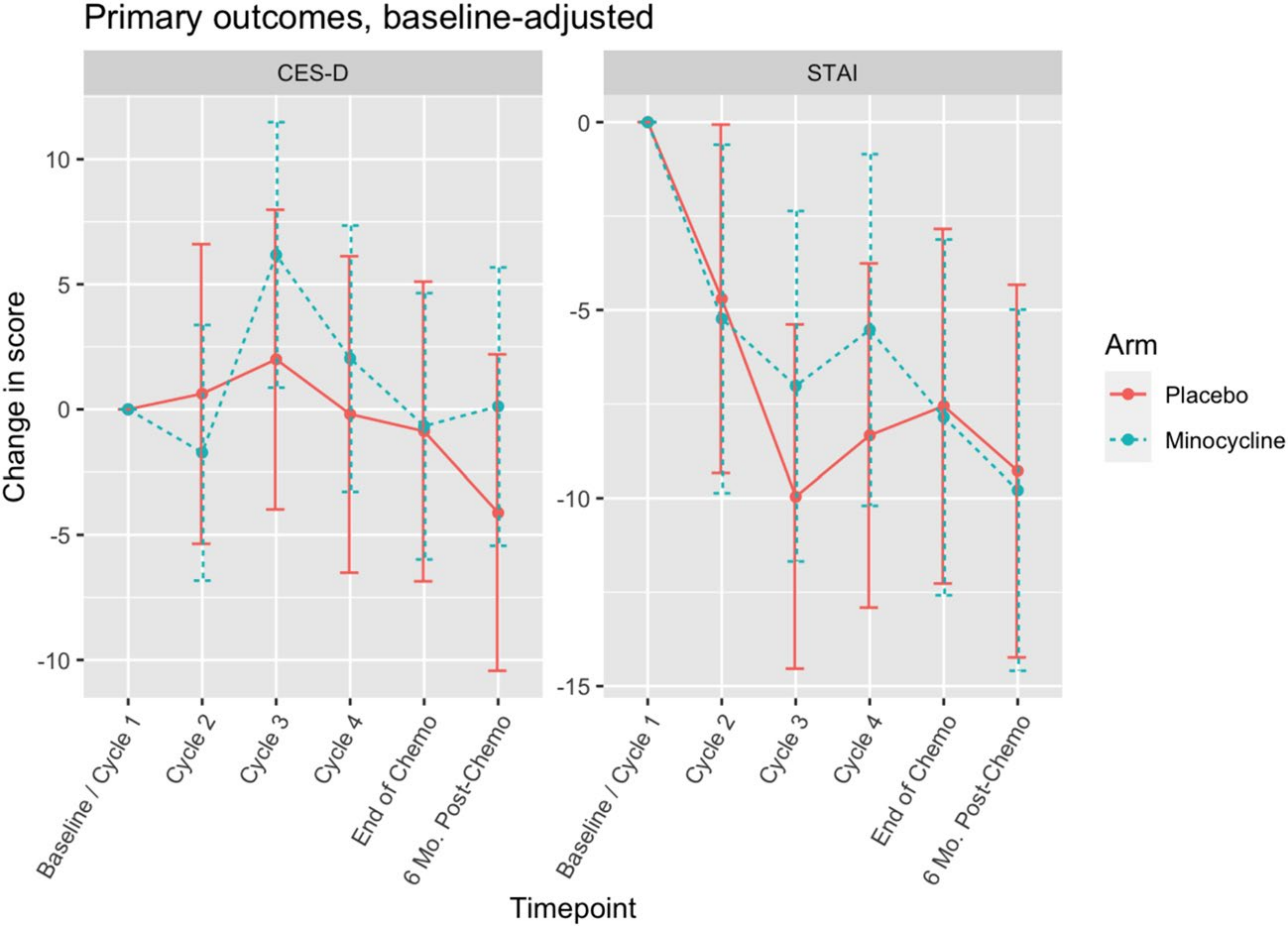
Changes in CES-D and STAI in women with breast cancer undergoing chemotherapy treated with placebo or minocycline. The Epidemiologic Studies Depression Scale (CES-D) was used to measure self-reported depression, and the State-Trait Anxiety Inventory (STAI) was used to measure self-reported anxiety. Baseline-adjusted scores were used. Baseline/Cycle 1: Prior to the 1st cycle of chemotherapy; Cycle 2: Prior to the 2nd cycle of chemotherapy; Cycle 3: Prior to the 3rd cycle of chemotherapy; Cycle 4: Prior to the 4th cycle of chemotherapy; End of Chemo: Within 2–3 weeks of last chemotherapy; 6-Mo. Post-Chemo: 6 months after cycle 4 chemotherapy

The mean raw scores of CES-D at each time point are shown in Table 2. The mean change in CES-D from baseline to end of chemotherapy was 0.57 (SD 7.95) in the minocycline group versus − 3.18 (SD 7.25) in the placebo group. Lower CES-D scores suggest fewer depressive symptoms. After adjusting for baseline CES-D, there was no statistically significant difference in mean changes between groups from baseline to any cycle (Fig. 1a, *p* = 0.18–0.89). Sub-group analysis among those with depressive symptoms at baseline (CES-D > = 16, *n* = 4 in minocycline, 11 in placebo group) revealed a statistically significant difference between minocycline and placebo in mean CES-D change from baseline to 6-month post-chemo (− 18.25, 95% CI − 27.65 to − 8.85, *p* = 0.01) suggesting fewer depressive symptoms in the minocycline group. However, only four subjects symptomatic at baseline were randomly assigned to the minocycline group. No other significant differences were found at any other timepoints (*p* = 0.14–0.85). Among those not symptomatic at baseline (CES-D < 16, *n* = 21 in minocycline, 13 in placebo group), there was no statistically significant difference between minocycline and placebo in mean CES-D change from baseline to any cycle (*p* = 0.06–0.68).

**Table 2:** Mean raw scores of CES-D and STAI in women with breast cancer undergoing chemotherapy treated with placebo or minocycline at different timepoints

Affective behavior test	CES-D						STAI					
Timepoints	Minocycline			Placebo			Minocycline			Placebo		
	*n*	Mean	SD	*n*	Mean	SD	*n*	Mean	SD	*n*	Mean	SD
Baseline/Cycle 1	25	10.8	8.58	24	14.5	9.51	24	38.0	13.4	24	43.2	11.2
Cycle 2	25	9.80	6.1	24	11.3	9.53	25	34.8	10.2	24	36.6	13.4
Cycle 3	24	11.4	10.1	24	11.6	9.30	24	33.0	9.30	24	31.5	12.7
Cycle 4	24	11.0	8.28	24	13.2	9.73	24	35.7	14.4	24	33.2	10.3
End of chemo	23	10.4	7.08	22	11.4	9.25	23	32.3	10.6	22	33.7	13.5
6-Mo. Post-chemo	22	7.95	6.82	19	10.9	8.24	22	30.5	15.2	19	31.5	10.5

The Epidemiologic Studies Depression Scale (CES-D) was used to measure self-reported depression, and the State-Trait Anxiety Inventory (STAI) was used to measure self-reported anxiety. Baseline/Cycle 1: Prior to the 1st cycle of chemotherapy; Cycle 2: Prior to the 2nd cycle of chemotherapy; Cycle 3: Prior to the 3rd cycle of chemotherapy; Cycle 4: Prior to the 4th cycle of chemotherapy; End of Chemo: Within 2–3 weeks of last chemotherapy; 6-Mo. Post-Chemo: 6 months after cycle 4 chemotherapy

The mean raw scores of STAI at each time point are shown in Table 2. The mean change in STAI from baseline to end of chemotherapy was − 5.86 (SD 13.13) in the minocycline group versus − 9.41 (SD 15.72) in the placebo group. A lower STAI score suggests a lower anxiety level [54]. After adjusting for baseline STAI, there was no statistically significant difference in STAI mean changes between groups from baseline to any cycle (Fig. 1b, p = 0.38–0.93). Sub-group analysis among those symptomatic for anxiety at baseline (STAI > = 40, *n* = 13 in minocycline, 16 in placebo group) found no statistically significant difference between minocycline and placebo in mean STAI change from baseline to any cycle (*p* = 0.48–0.76). Among those not symptomatic at baseline (STAI < 40, *n* = 11 in minocycline, 8 in placebo group), there was no statistically significant difference between minocycline and placebo in mean STAI change from baseline to any cycle (*p* = 0.31–0.96).

### Mean scores and changes in cognitive scores

**Fig. 2:**
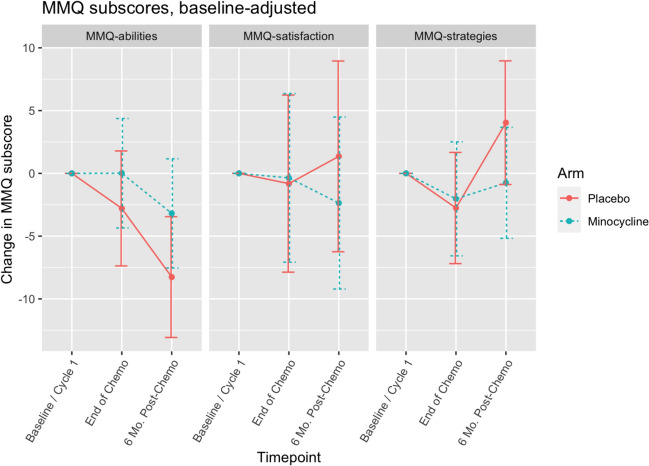
Changes in Multifactorial Memory Questionnaire (MMQ) scores in women with breast cancer undergoing chemotherapy treated with placebo or minocycline. The Multifactorial Memory Questionnaire (MMQ) has three sub-indices: MMQ-ability, MMQ-satisfaction, and MMQ-strategy. Higher MMQ-satisfaction and MMQ-ability scores and lower MMQ-strategy scores suggest better memory performance. Baseline-adjusted scores were used. Baseline /Cycle 1: Prior to the first cycle of chemotherapy; End of Chemo: Within 2–3 weeks after last chemotherapy; 6-Mo. Post-Chemo: 6 months after cycle 4 chemotherapy

The mean raw scores of MMQ at each timepoint are shown in Table 3. Changes in self-reported memory measured by MMQ are shown in Fig. 2. The mean changes in MMQ-satisfaction, MMQ-abilities, and MMQ-strategies scores from baseline to end of chemotherapy was 0.09 (SD 12.36), 1 (SD 10.79), and − 2.58 (SD 10.78) in the minocycline group, versus − 1.35 (SD 9.67), − 3.89 (SD 8.79), and − 2.2 (SD 13.25) in the placebo group. Adjusting for baseline MMQ scores, there was no statistically significant difference in mean changes between groups from baseline to any cycle (Fig. 2, *p* = 0.18–0.89 for MMQ-satisfaction scores, *p* = 0.48–0.93 for MMQ-abilities, *p* = 0.16–0.82 for MMQ-strategies).

**Table 3:** Mean raw scores of MMQ and BRIEF A in women with breast cancer undergoing chemotherapy treated with placebo or minocycline at different timepoints

Timepoints	Cognitive tests	Minocycline			Placebo		
		*n*	Mean	SD	*n*	Mean	SD
Baseline	MMQ-Satisfaction	22	48.0	13.1	20	53.6	14.6
End of chemo		23	47.8	15.0	22	51.9	14.5
6-month post-chemotherapy		22	45.8	13.1	19	53.3	31.3
Baseline	MMQ-Abilities	22	51.5	12.7	20	57.2	9.7
End of chemo		23	51.8	12.8	22	53.2	11.8
6-month post-chemotherapy		22	49.0	12.1	19	48.7	14.9
Baseline	MMQ-Strategies	21	34.5	12.9	20	31.8	14.9
End of chemo		23	32.9	11.4	22	30.5	10.2
6-month post-chemotherapy		22	33.3	13.2	19	37.9	15.0
Baseline	BRIEF-A-BRI	21	52.3	7.6	23	56.1	12.9
End of chemo		23	55.5	9.0	22	54.4	9.5
6-month post-chemotherapy		22	55.1	9.6	19	54.8	11.9
Baseline	BRIEF A-MI	23	52.0	10.3	21	54.5	10.9
End of chemo		23	54.4	11.3	22	51.6	11.3
6-month post-chemotherapy		22	55.1	9.8	19	52.2	13.2
Baseline	BRIEF A-GEC	23	104.0	17.2	21	111.0	21.9
End of chemo		23	110.0	19.5	22	106.0	20.3
6-month post-chemotherapy		22	110.0	18.8	19	107.0	24.9

The Multifactorial Memory Questionnaire (MMQ) has three sub-indices: MMQ-ability, MMQ-satisfaction, and MMQ-strategy. Higher MMQ-satisfaction and MMQ-ability scores and lower MMQ-strategy scores suggest better memory performance. Baseline /Cycle 1: Prior to the first cycle of chemotherapy; End of Chemo: Within 2–3 weeks after last chemotherapy; 6-Mo. Post-Chemo: 6 months after cycle 4 chemotherapy. The Behavior Rating Inventory of Executive Function-Adult Version (BRIEF A) has two sub-indices: the Behavioral Regulation Index (BRI, questions 1–38) and the Metacognitive Index (MI, questions 39–75). The sum of the two sub-indices yields Global Executive Composite (Total). For the total score GEC and the sub-indices MI and BRI, lower scores suggest better cognitive and executive functions

**Fig. 3:**
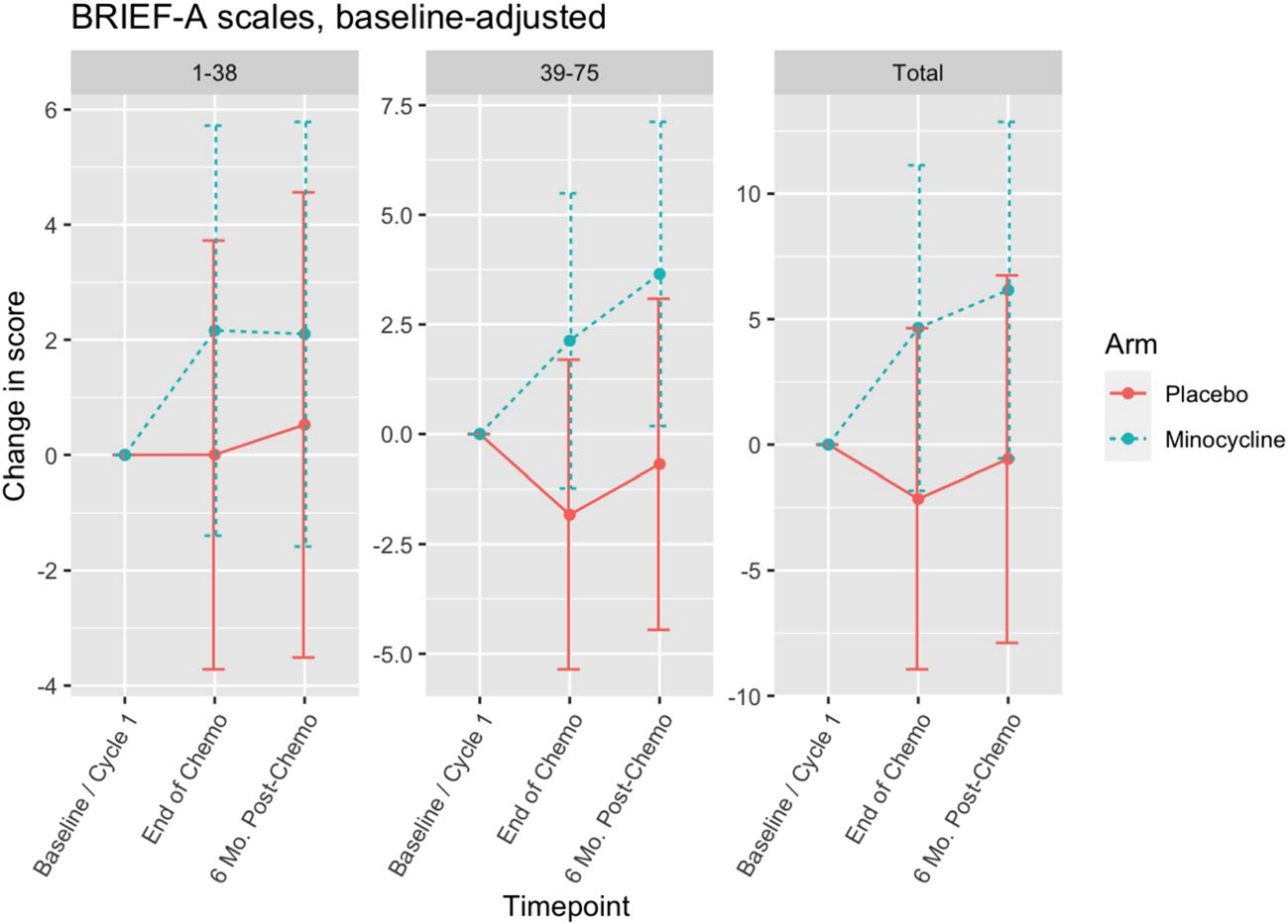
Changes in Behavior Rating Inventory of Executive Function-Adult Version (BRIEF A) scores in women with breast cancer undergoing chemotherapy treated with placebo or minocycline. The Behavior Rating Inventory of Executive Function-Adult Version (BRIEF A) has two sub-indices: the Behavioral Regulation Index (BRI, questions 1–38) and the Metacognitive Index (MI, questions 39–75). The sum of the two sub-indices yields Global Executive Composite (Total). For the total score GEC and the sub-indices MI and BRI. Lower scores suggest better cognitive and executive functions. Baseline-adjusted scores were used. Baseline/ Cycle 1: Prior to the first cycle of chemotherapy; End Chemo: Within 2–3 weeks after last chemotherapy; 6-Mo. Post-Chemo: 6 months after cycle chemotherapy

The mean raw scores of BRIEF A at each timepoint are shown in Table 3. Changes in self-reported executive function measured by BRIEF A are shown in Fig. 3. The mean changes in BRIEF A part 1 (BRI), BRIEF A part 2 (MI), and BRIEF A total from baseline to end of chemotherapy was 3.22 (SD 8.31), 2.39 (SD 9.83), and 5.61 (SD 17.04) for the minocycline group and − 1.05 (SD 12.67), − 2.24 (SD 8.65), and − 3.29 (SD 18.77) for the placebo group. Adjusting for baseline BRIEF A scores, there was no statistically significant difference in mean changes between groups from baseline to any cycle (Fig. 3, *p* = 0.42–0.58 for BRIEF A (1–38), *p* = 0.1–0.12 for BRIEF A part 2 (MI), *p* = 0.16–0.19 for BRIEF A total).

### Peripheral inflammation

The mean values of five inflammatory markers measured at Baseline/Cycle 1, Cycle 4, and 6 M are shown in Table 4. The mean changes in serum biomarkers of inflammation by randomization arms are detailed in Supplementary Table 2.

**Table 4:** Mean values of inflammatory markers in women with breast cancer undergoing chemotherapy treated with placebo or minocycline at different timepoints

Timepoints	Inflammatory markers (pg/ml)	Minocycline			Placebo		
		*n*	Mean	SD	*n*	Mean	SD
Baseline	IL-1β	26	0.076	0.044	21	0.061	0.032
Cycle 4		17	0.061	0.036	20	0.060	0.035
6-month post-chemotherapy		18	0.069	0.039	15	0.063	0.032
Baseline	IL-6	26	0.88	0.57	21	1.07	1.05
Cycle 4		17	1.07	1.01	20	1.18	0.86
6-month post-chemotherapy		18	1.40	1.06	15	1.79	1.66
Baseline	IL-8	26	11.0	7.56	21	7.90	3.43
Cycle 4		17	6.48	2.99	20	8.13	6.25
6-month post-chemotherapy		18	8.92	4.57	15	11.30	10.00
Baseline	TNF-α	26	2.14	0.89	21	2.52	2.81
Cycle 4		17	2.00	0.59	20	2.34	1.20
6-month post-chemotherapy		18	2.82	1.86	15	2.30	0.92
Baseline	TNFR-II	26	7715	2644	21	7166	2279
Cycle 4		17	8939	2507	20	8748	3878
6-month post-chemotherapy		18	8695	4698	15	7452	1932

Interleukin-1beta (IL-1b), interleukin-6 (IL-6), interleukin-8 (IL-8), tumor necrosis factor-alpha (TNF-α), and tumor necrosis factor receptor 2 (TNF-RII). All values were baseline adjusted. Baseline/Cycle 1: before the first cycle of chemotherapy; Cycle 4: before the fourth cycle of chemotherapy; 6-Mo. Post-Chemo: 6 months after cycle 4 chemotherapy

**Fig. 4:**
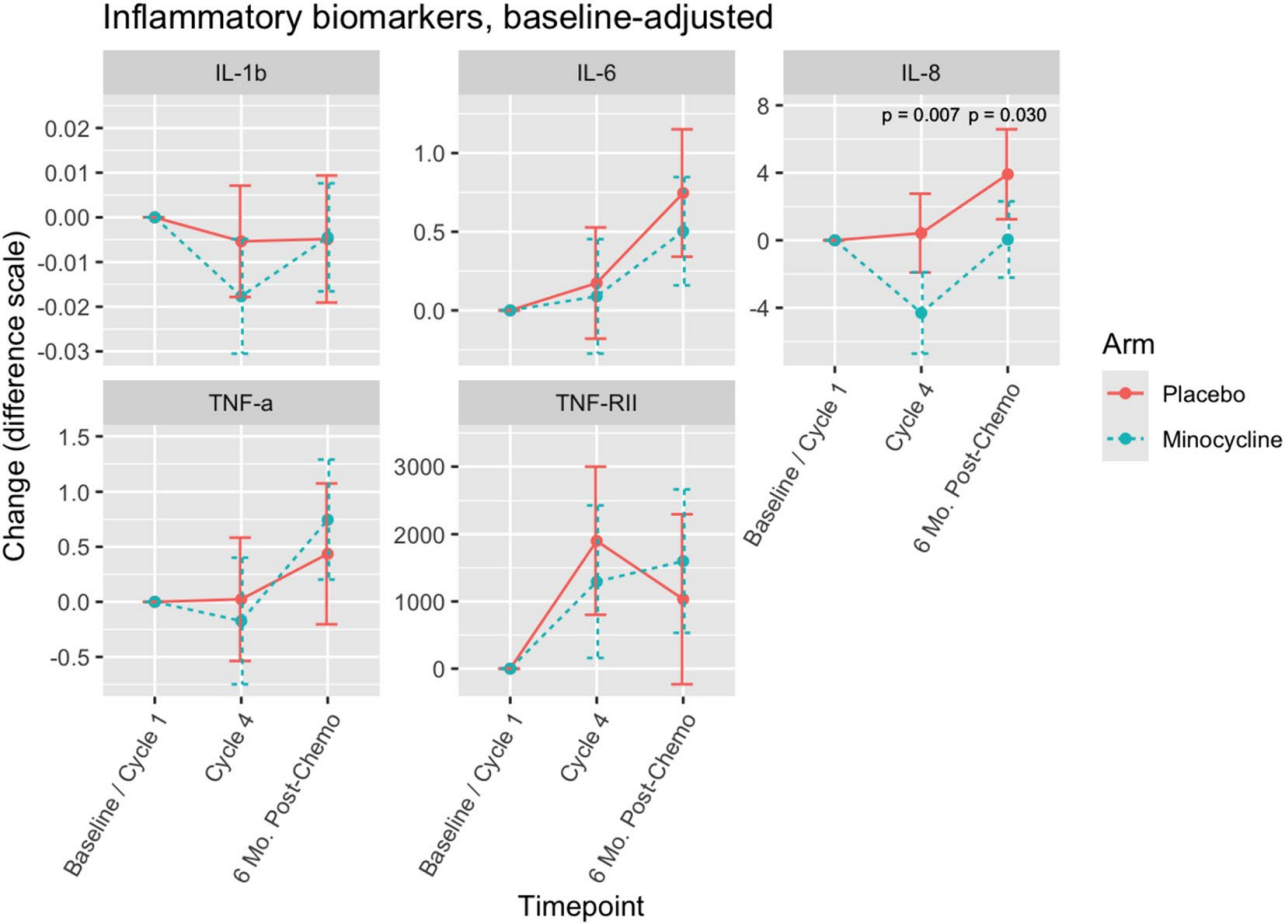
Changes in serum inflammatory biomarkers in women with breast cancer undergoing chemotherapy treated with placebo or minocycline. Interleukin-1beta (IL-1b), interleukin-6 (IL-6), interleukin-8 (IL-8), tumor necrosis factor-alpha (TNF-α), and tumor necrosis factor receptor 2 (TNF-RII). All values were baseline-adjusted. Baseline/Cycle 1: before the first cycle of chemotherapy; Cycle 4: before the fourth cycle of chemotherapy; 6-Mo. Post-Chemo: 6 months after cycle 4 chemotherapy

Apart from IL-8, there were no significant differences between randomized groups in mean changes in biomarkers over the course of chemotherapy (Fig. 4a–e, Supplementary Table 2). The mean change in IL-8 from baseline to cycle 4 was − 4.65 (SD 7.02) pg/ml in the minocycline group versus 0.79 (SD 5.59) pg/ml in the placebo group. Adjusting for baseline IL-8, there was a significant difference in mean changes between groups from baseline [Fig. 4c, baseline to cycle 4 (minocycline − 4.303, placebo 0.426), *p* = 0.007, and from baseline to 6-month post-chemotherapy (minocycline 0.0473, placebo 3.913), *p* = 0.030] such that minocycline treatment ameliorated the increase in IL-8 compared to placebo. Changes in inflammatory biomarkers in the entire sample of participants (baseline-adjusted) are shown in Supplementary Fig. 2. There were significant decreases in IL-1b (*p* = 0.010) and IL-8 (*p* = 0.020) and increases in TNF-RII (*p* < 0.001) from baseline to cycle 4, as well as significant increases in IL-6 (*p* = 0.008), IL-8 (*p* = 0.002), and TNF-α (*p* = 0.030) from cycle 4 to 6-month post-chemotherapy.

## Discussion

Minocycline is a second-generation, broad-spectrum tetracycline antibiotic [27, 28]. Many preclinical and clinical studies have shown that minocycline has anti-inflammatory properties [24–29]. Additionally, preclinical data suggest that minocycline may protect against neurodegenerative and neuroinflammatory diseases [30–32]. In this pilot randomized controlled trial (RCT), we investigated the effect of minocycline versus placebo treatment on affective disorders, cognitive function, and peripheral inflammation in this cohort of women undergoing chemotherapy for breast cancer.

CES-D increased by 0.57 (SD 7.95) from baseline to end of chemotherapy in the minocycline group and decreased by 3.18 (SD 7.25) in the placebo group. A lower CES-D score suggests lower depressive symptoms. STAI reduced from baseline to end of chemotherapy by 5.86 (SD 13.13) in the minocycline group, while it decreased by 9.41 (SD 15.72) in the placebo group. A decreased STAI score suggests a reduced anxiety level [18]. Results of this study demonstrate that oral minocycline (100-mg BID) did not have a significant effect on depression measured by CES-D or anxiety measured by STAI over the course of four cycles of chemotherapy when compared to matched placebo. In contrast, in one RCT with male schizophrenia patients aged 18–65 years, researchers found improvement in anxiety and depressive symptoms when 100-mg BID minocycline was administered [55]. Another RCT with male participants (mean age = 35–50) also found that adjunctive minocycline (100-mg BID–200-mg BID) improved symptoms of treatment-resistant depression [56]. However, the differences in our study results might be related to different disease mechanisms, demographic characteristics, such as sex and age of participants, and the differences in severity of depressive symptoms in the studies. Similar to our results, a few RCTs with patients with treatment-resistant depression and bipolar depression suggested that minocycline did not ameliorate depressive symptoms compared to placebo groups [57, 58].

One recently published RCT by Nettis et al. evaluated minocycline’s effects in women with treatment-resistant depression. Similar to our study, they did not find significant differences in changes in depression when examining the entire cohort [59]. Interestingly, after stratifying the participants by C-Reactive Protein (CRP) level, they did find that minocycline attenuated depressive symptoms in the high inflammation group (CRP > 3 mg) [59]. Considering that minocycline might be more effective in BC survivors who were symptomatic for depressive or anxiety symptoms at baseline, prior to chemotherapy, the differences in CES-D and STAI scores were evaluated in those symptomatic and non-symptomatic subgroups. Our results suggest that BC survivors symptomatic for depression prior to chemotherapy may benefit from minocycline treatment. However, this data is very preliminary as only four participants with CES-D scores indicative of depression were randomized to the minocycline group. Therefore, such effects need to be further evaluated in larger studies.

Adjusting for baseline, none of the changes in self-reported assessments, including MMQ-satisfaction, MMQ-abilities, and MMQ-strategies, BRIEF A-BRI, BRIEF A-MI, and BRIEF A-GEC scores, were significant. Minocycline did not have a significant effect on self-reported memory or executive function changes during chemotherapy treatment in our cohort. Although there is minimal research on the effect of minocycline on cognitive function in breast cancer, one preclinical study has identified minocycline as a potential neuroprotectant for cancer-related cognitive impairment (CRCI) using a xenograft model of triple-negative breast cancer while co-administered with doxorubicin [34]. However, the clinical effect of minocycline on non-cancer-related cognitive disorders and neurodegenerative diseases has been disappointing [60–63], as cited in a recent review [64].

Although serum inflammatory markers increased overall throughout chemotherapy, apart from IL-8, we found no significant differences in biomarkers between minocycline and placebo groups. IL-8 increased more in the placebo group compared to the minocycline group from baseline to any cycles after adjusting for baseline IL-8 level. IL-8 is a cytokine that can be produced by various tissues and secreted by multiple types of cells and is involved in mitogenesis, inflammation, leukocyte activation, and calcium homeostasis [65, 66]. Unlike other cytokines measured, IL-8 is a chemoattractant for neutrophils [65, 66]. Although inflammation is commonly believed to be associated with depression and anxiety [32, 67, 68], recent studies have shown that IL-8 might have neuroprotective properties [69] and might be associated with better depression treatment outcomes [70–73]. In a prospective cohort study, Irwin and colleagues have found that elevated IL-8 was associated with lower severity of depressive symptoms, favorable treatment responses, and a decreased rate of new and recurrent major depression in breast cancer survivors [70]. Taken together, these studies suggest that women with lower baseline IL-8 levels might be better candidates for anti-depressant therapy and increases in IL-8 might be related to decreases in affective symptoms. Similar to our results, Nettis et al. found that women treated with minocycline for treatment-resistant depression did not significantly differ from the placebo group in changes in IL-6, IL-1β, and TNF-α, after four weeks of treatment [59]. However, Nettis et al. did not find significant differences in the mean changes in IL-8 levels. This difference might be caused by the different pathologies and populations studied.

### Strengths and limitations

Strengths of this pilot study include the randomized controlled design, the inclusion of multiple assessments of affective disorders and cognitive function, and the ability to explore potential mechanisms through the measurement of multiple biomarkers of inflammation. Additionally, retention of nearly 95% of participants through primary outcome measurements is a strength and points to the value that participants placed on research addressing mitigation of affective and cognitive issues that impact quality of life. It is also notable that minocycline was very well tolerated, which may have contributed to high retention rates. However, this study had several limitations. First, we included women who did not display symptoms of depression and anxiety at baseline in this study. This could have reduced our ability to detect any potential benefit of minocycline on these symptoms. Additionally, participants received various chemotherapy regimens, and the effect of minocycline might be altered based on the chemotherapy treatment. For example, a previous study found that minocycline might protect against AC-induced neurological damage [34], but approximately, 51% of women in our study were treated with non-AC-based regimens, which may have influenced our results. Moreover, other factors, such as social support, BMI, and age, might also alter affective symptoms and cognitive function [74, 75]. Our sample size was not powered to investigate differences in outcomes related to these variables. Our sample consisted almost entirely of non-Hispanic white women, which limits the generalizability of our results. Additionally, our outcome assessment tools may not have been sensitive enough to detect all meaningful differences. For example, some of the changes in depression might be domain specific [76], and the subscales of CES-D were not measured in our study. Also, as pointed out by Kuhlman and colleagues, CES-D measures depression based on factors easily modified by other factors, such as sleep and appetite, and those measurements can fluctuate during a depression episode [76]. In addition, although STAI is a validated tool for measuring anxiety, HADS is the most commonly used depression screening questionnaire for people with cancer [41, 77]. Finally, cognitive assessments were self-reported and did not include objective cognitive tests. Other questionnaires and more objective tests may have strengthened the study. Future studies could consider adaptive designs with even greater patient involvement focusing on patients with early evidence of affective or cognitive symptoms, using minocycline or other anti-inflammatory interventions.

## Conclusion

Overall, administering minocycline did not mitigate changes in symptoms of anxiety or depression, self-reported executive function or memory, or the inflammatory profile of breast cancer patients undergoing chemotherapy. Future research should determine if specific subgroups of patients, such as those symptomatic for depression or anxiety at baseline or those with high baseline levels of inflammation, may benefit from minocycline treatment during chemotherapy.

## Supplementary Information

Below is the link to the electronic supplementary material.

Supplementary file1 (DOCX 267 kb)

## Data Availability

Data available from the corresponding author upon request.
