# Supplementary material for: Effect of minocycline on changes in affective behaviors, cognitive function, and inflammation in breast cancer survivors undergoing chemotherapy: a pilot randomized controlled trial
Source: Breast Cancer Res Treat. 2024 Aug 14;208(3):605–17. doi: 10.1007/s10549-024-07457-w (PMC11522141; doi:10.1007/s10549-024-07457-w)
Supplement: Supplementary file 1 — Supplementary file1 (DOCX 267 kb) [file 10549_2024_7457_MOESM1_ESM.docx]

**Supplementary Table 1**. Participants Self-reporting Adverse Event (Any Grade) in Breast Cancer Survivors Undergoing Chemotherapy Randomized to Minocycline and Placebo Group

| Adverse Event | MINOCYCLINE (n, %) | PLACEBO (n, %) | p.value |
| --- | --- | --- | --- |
| Any | 25 (89.3%) | 24 (85.7%) | 1.00 |
| Hypertension | 17 (60.7%) | 19 (67.9%) | 0.80 |
| Nausea | 16 (57.1%) | 17 (60.7%) | 1.00 |
| Fatigue | 15 (53.6%) | 20 (71.4%) | 0.30 |
| Anemia | 15 (53.6%) | 17 (60.7%) | 0.80 |
| Hyperglycemia | 14 (50%) | 17 (60.7%) | 0.60 |
| Constipation | 10 (35.7%) | 10 (35.7%) | 1.00 |
| Dizziness | 10 (35.7%) | 9 (32.1%) | 1.00 |
| Headache | 9 (32.1%) | 13 (46.4%) | 0.40 |
| Bone pain | 9 (32.1%) | 6 (21.4%) | 0.50 |
| Diarrhea | 8 (28.6%) | 18 (64.3%) | 0.02 |
| Dyspnea | 8 (28.6%) | 10 (35.7%) | 0.80 |
| Mucositis oral | 7 (25%) | 13 (46.4%) | 0.20 |
| Peripheral motor neuropathy | 7 (25%) | 7 (25%) | 1.00 |
| Anorexia | 7 (25%) | 6 (21.4%) | 1.00 |
| Vomiting | 6 (21.4%) | 12 (42.9%) | 0.20 |
| Insomnia | 6 (21.4%) | 8 (28.6%) | 0.80 |
| Pain | 6 (21.4%) | 6 (21.4%) | 1.00 |
| Rash maculopapular | 6 (21.4%) | 6 (21.4%) | 1.00 |
| Cough | 6 (21.4%) | 5 (17.9%) | 1.00 |
| Edema limbs | 5 (17.9%) | 12 (42.9%) | 0.08 |
| Hot flashes | 5 (17.9%) | 2 (7.1%) | 0.40 |
| Back pain | 4 (14.3%) | 3 (10.7%) | 1.00 |
| Dysgeusia | 4 (14.3%) | 3 (10.7%) | 1.00 |
| Fever | 4 (14.3%) | 3 (10.7%) | 1.00 |
| Paresthesia | 4 (14.3%) | 0 (0%) | 0.10 |
| Epistaxis | 3 (10.7%) | 4 (14.3%) | 1.00 |
| Alopecia | 3 (10.7%) | 1 (3.6%) | 0.60 |
| Anxiety | 2 (7.1%) | 7 (25%) | 0.10 |
| Platelet count decreased | 2 (7.1%) | 5 (17.9%) | 0.40 |
| Depression | 2 (7.1%) | 4 (14.3%) | 0.70 |
| Allergic rhinitis | 2 (7.1%) | 2 (7.1%) | 1.00 |
| Dry mouth | 2 (7.1%) | 2 (7.1%) | 1.00 |
| Hypokalemia | 2 (7.1%) | 1 (3.6%) | 1.00 |
| Skin infection | 2 (7.1%) | 1 (3.6%) | 1.00 |
| Hypoglycemia | 2 (7.1%) | 0 (0%) | 0.50 |
| Irritability | 2 (7.1%) | 0 (0%) | 0.50 |
| Watering eyes | 1 (3.6%) | 4 (14.3%) | 0.40 |
| Arthralgia | 1 (3.6%) | 3 (10.7%) | 0.60 |
| Peripheral sensory neuropathy | 1 (3.6%) | 3 (10.7%) | 0.60 |
| Breast pain | 1 (3.6%) | 2 (7.1%) | 1.00 |
| Dyspepsia | 1 (3.6%) | 2 (7.1%) | 1.00 |
| Eye disorders - Other, specify | 1 (3.6%) | 2 (7.1%) | 1.00 |
| Pruritus | 1 (3.6%) | 2 (7.1%) | 1.00 |
| Rash acneiform | 1 (3.6%) | 2 (7.1%) | 1.00 |
| Abdominal pain | 1 (3.6%) | 1 (3.6%) | 1.00 |
| Alanine aminotransferase increased | 1 (3.6%) | 1 (3.6%) | 1.00 |
| Alkaline phosphatase increased | 1 (3.6%) | 1 (3.6%) | 1.00 |
| Arthritis | 1 (3.6%) | 1 (3.6%) | 1.00 |
| Bruising | 1 (3.6%) | 1 (3.6%) | 1.00 |
| CD4 lymphocytes decreased | 1 (3.6%) | 1 (3.6%) | 1.00 |
| Flushing | 1 (3.6%) | 1 (3.6%) | 1.00 |
| Hypoalbuminemia | 1 (3.6%) | 1 (3.6%) | 1.00 |
| Myalgia | 1 (3.6%) | 1 (3.6%) | 1.00 |
| Nail discoloration | 1 (3.6%) | 1 (3.6%) | 1.00 |
| Nasal congestion | 1 (3.6%) | 1 (3.6%) | 1.00 |
| Non-cardiac chest pain | 1 (3.6%) | 1 (3.6%) | 1.00 |
| Skin and subcutaneous tissue disorders - Other, specify | 1 (3.6%) | 1 (3.6%) | 1.00 |
| Urinary frequency | 1 (3.6%) | 1 (3.6%) | 1.00 |
| Urinary tract infection | 1 (3.6%) | 1 (3.6%) | 1.00 |
| Agitation | 1 (3.6%) | 0 (0%) | 1.00 |
| Dry skin | 1 (3.6%) | 0 (0%) | 1.00 |
| Dysphagia | 1 (3.6%) | 0 (0%) | 1.00 |
| Facial pain | 1 (3.6%) | 0 (0%) | 1.00 |
| Gastritis | 1 (3.6%) | 0 (0%) | 1.00 |
| Generalized muscle weakness | 1 (3.6%) | 0 (0%) | 1.00 |
| Hemoglobin increased | 1 (3.6%) | 0 (0%) | 1.00 |
| Hypocalcemia | 1 (3.6%) | 0 (0%) | 1.00 |
| Hyponatremia | 1 (3.6%) | 0 (0%) | 1.00 |
| Hypotension | 1 (3.6%) | 0 (0%) | 1.00 |
| Infections and infestations - Other, specify | 1 (3.6%) | 0 (0%) | 1.00 |
| Irregular menstruation | 1 (3.6%) | 0 (0%) | 1.00 |
| Lip infection | 1 (3.6%) | 0 (0%) | 1.00 |
| Memory impairment | 1 (3.6%) | 0 (0%) | 1.00 |
| Mucosal infection | 1 (3.6%) | 0 (0%) | 1.00 |
| Neck pain | 1 (3.6%) | 0 (0%) | 1.00 |
| Pain in extremity | 1 (3.6%) | 0 (0%) | 1.00 |
| Papulopustular rash | 1 (3.6%) | 0 (0%) | 1.00 |
| Postnasal drip | 1 (3.6%) | 0 (0%) | 1.00 |
| Renal calculi | 1 (3.6%) | 0 (0%) | 1.00 |
| Restlessness | 1 (3.6%) | 0 (0%) | 1.00 |
| Sinusitis | 1 (3.6%) | 0 (0%) | 1.00 |
| Skin hyperpigmentation | 1 (3.6%) | 0 (0%) | 1.00 |
| Sore throat | 1 (3.6%) | 0 (0%) | 1.00 |
| Urinary incontinence | 1 (3.6%) | 0 (0%) | 1.00 |
| Vaginal pain | 1 (3.6%) | 0 (0%) | 1.00 |
| Hemorrhoids | 0 (0%) | 4 (14.3%) | 0.10 |
| Confusion | 0 (0%) | 3 (10.7%) | 0.20 |
| Upper respiratory infection | 0 (0%) | 3 (10.7%) | 0.20 |
| Blurred vision | 0 (0%) | 2 (7.1%) | 0.50 |
| Dehydration | 0 (0%) | 2 (7.1%) | 0.50 |
| Dry eye | 0 (0%) | 2 (7.1%) | 0.50 |
| Edema face | 0 (0%) | 2 (7.1%) | 0.50 |
| Localized edema | 0 (0%) | 2 (7.1%) | 0.50 |
| Lymphocyte count decreased | 0 (0%) | 2 (7.1%) | 0.50 |
| Palmar-plantar erythrodysesthesia syndrome | 0 (0%) | 2 (7.1%) | 0.50 |
| Respiratory, thoracic and mediastinal disorders - Other, specify | 0 (0%) | 2 (7.1%) | 0.50 |
| Weight gain | 0 (0%) | 2 (7.1%) | 0.50 |
| Akathisia | 0 (0%) | 1 (3.6%) | 1.00 |
| Bloating | 0 (0%) | 1 (3.6%) | 1.00 |
| Bronchial infection | 0 (0%) | 1 (3.6%) | 1.00 |
| Bullous dermatitis | 0 (0%) | 1 (3.6%) | 1.00 |
| Eye pain | 0 (0%) | 1 (3.6%) | 1.00 |
| Fall | 0 (0%) | 1 (3.6%) | 1.00 |
| Floaters | 0 (0%) | 1 (3.6%) | 1.00 |
| Gastroesophageal reflux disease | 0 (0%) | 1 (3.6%) | 1.00 |
| Hemorrhoidal hemorrhage | 0 (0%) | 1 (3.6%) | 1.00 |
| Hyperhidrosis | 0 (0%) | 1 (3.6%) | 1.00 |
| Hyperkalemia | 0 (0%) | 1 (3.6%) | 1.00 |
| Laryngeal inflammation | 0 (0%) | 1 (3.6%) | 1.00 |
| Lymphedema | 0 (0%) | 1 (3.6%) | 1.00 |
| Menorrhagia | 0 (0%) | 1 (3.6%) | 1.00 |
| Muscle weakness lower limb | 0 (0%) | 1 (3.6%) | 1.00 |
| Scalp pain | 0 (0%) | 1 (3.6%) | 1.00 |
| Sepsis | 0 (0%) | 1 (3.6%) | 1.00 |
| Tinnitus | 0 (0%) | 1 (3.6%) | 1.00 |
| Weight loss | 0 (0%) | 1 (3.6%) | 1.00 |

The only statistically significant difference in adverse events between randomized arms is a lower incidence in diarrhea in the minocycline arm (28.6% versus 64.3%, p = 0.02).

**Supplementary Table 2.** Serum Inflammatory Biomarker Levels in Breast Cancer Survivors Undergoing Chemotherapy Randomized to Placebo and Minocycline Groups

| Inflammatory Biomarkers (pg/ml) | Baseline to Cycle 4 (crude) | | | | p-value (baseline-adjusted) |
| --- | --- | --- | --- | --- | --- |
|  | Minocycline | | Placebo | |  |
|  | Mean | SD | Mean | SD |  |
| IL-6 | 0.09 | 0.92 | 0.16 | 0.83 | 0.745 |
| IL-8 | -4.65 | 7.02 | 0.79 | 5.59 | **0.007** |
| IL-1b | -0.02 | 0.04 | 0.00 | 0.04 | 0.181 |
| TNF-α | -0.16 | 0.73 | -0.15 | 2.52 | 0.632 |
| TNF-RII | 1283.42 | 2341.74 | 1904.57 | 3292.61 | 0.453 |

**Supplementary Fig. 1** Compliance with Minocycline and Placebo Doses in Breast Cancer Survivors Undergoing Chemotherapy


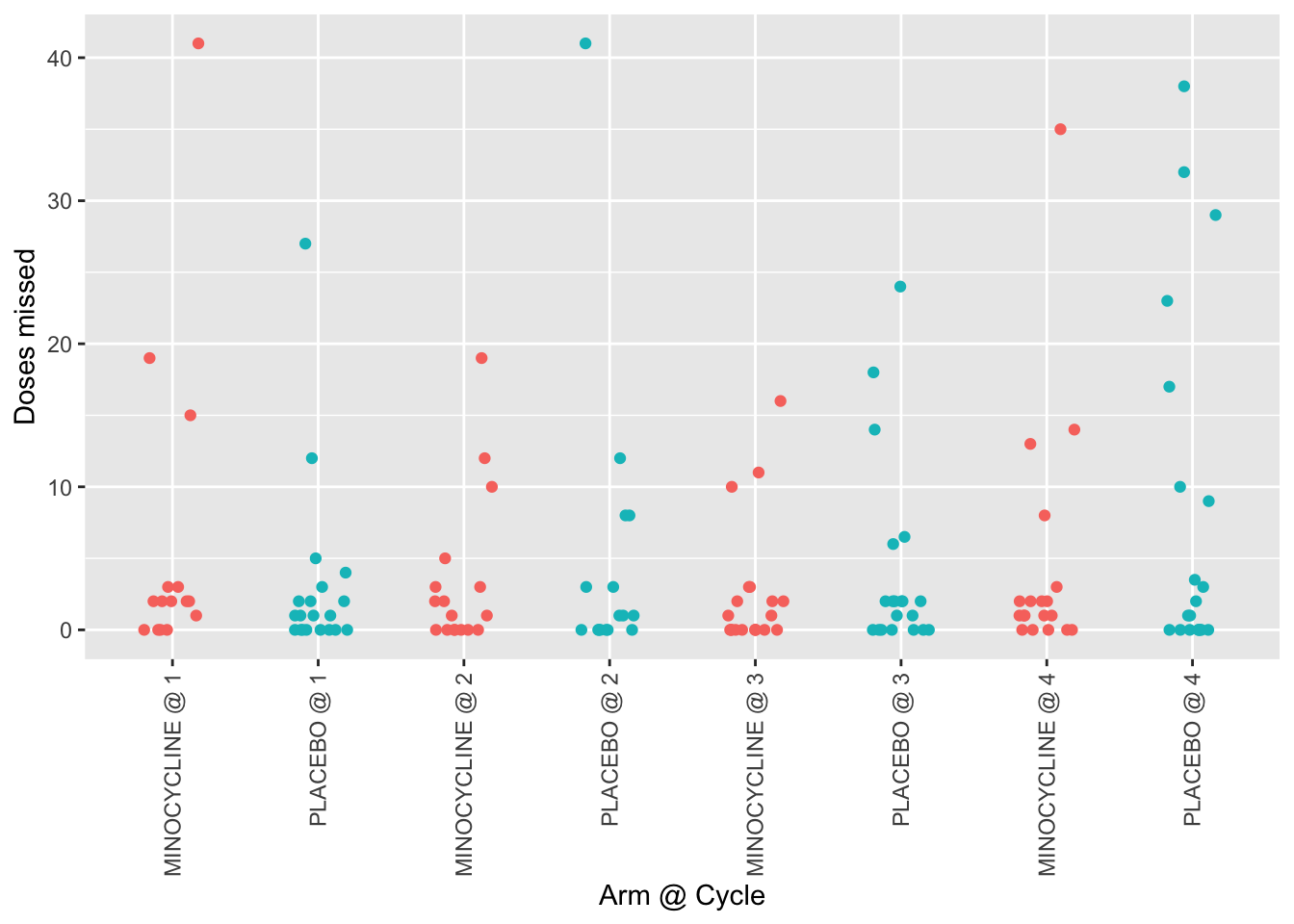


Minocycline Arm: Minocycline 100 mg BID. Placebo Arm: matched placebo capsule BID. Each dot represents one dose missed (e.g. one pill)

A Quasi-Poisson generalized linear model was used to test for differences in missed dose counts between randomized arms during each cycle. There were no statistically significant differences between groups at any cycle (p=0.21 to 0.59). X-axis timepoints are based on chemotherapy cycle durations of approximately 2-3 weeks: 1 = time between Cycle 1 and Cycle 2 chemotherapy; 2 = time between Cycle 2 and Cycle 3 chemotherapy; 3 = time between Cycle 3 and Cycle 4 chemotherapy; 4 = time between Cycle 4 chemotherapy and end of study drug administration (2-3 weeks post chemotherapy administration)

**Supplementary Fig. 2** Inflammatory Biomarkers (Baseline-Adjusted) in Breast Cancer Survivors Undergoing Chemotherapy: Both Randomization Arms Combined


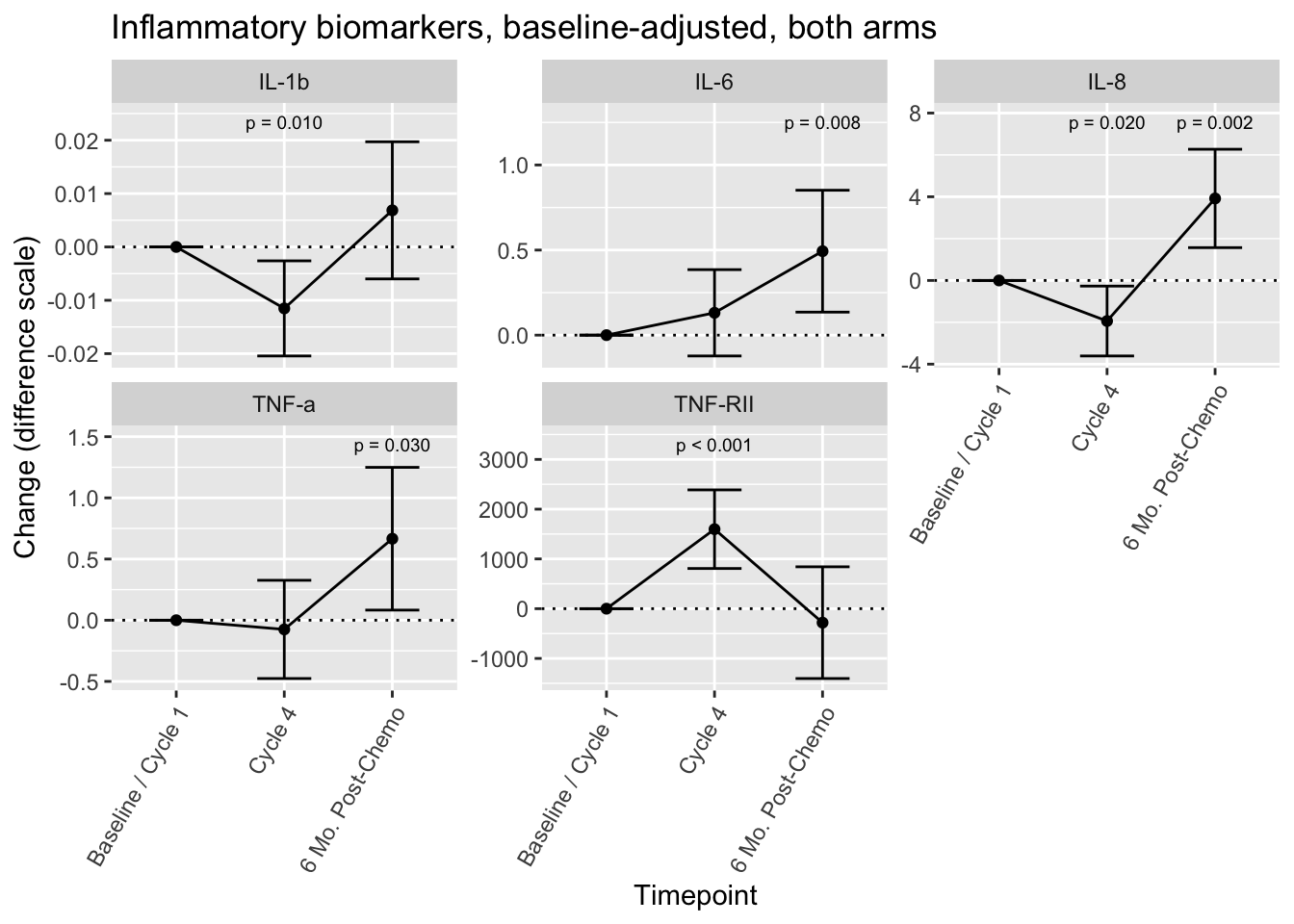
^[[1]](#footnote-2)^

1. Interleukin-1beta (IL-1b), interleukin-6 (IL-6), interleukin-8 (IL-8), tumor necrosis factor-alpha (TNF-α), and tumor necrosis factor receptor 2 (TNF-RII). All values were baseline-adjusted. Baseline/Cycle 1: before the first cycle of chemotherapy; Cycle 4: before the fourth cycle of chemotherapy; 6 Mo. Post-Chemo: 6 months after cycle 4 chemotherapy [↑](#footnote-ref-2)
